# Study on the mechanism by which Xuanfu Hua Tang increases sensitivity of hepatocellular carcinoma cells to sorafenib by antagonizing the Notch1 pathway through HIF-2α

**DOI:** 10.3389/fonc.2025.1552480

**Published:** 2025-05-15

**Authors:** Wenzhao Luo, Xian Li, Yiwan Shang, Zhen Chen, Yinglin Cui

**Affiliations:** ^1^ Henan University of Chinese Medicine, School of Basic Medicine (Zhongjing School), Zhengzhou, Henan, China; ^2^ Henan Province Hospital of Traditional Chinese Medicine, Department of Hepatobiliary and Spleen Stomach, Zhengzhou, Henan, China; ^3^ Henan University of Chinese Medicine, Academy of Chinese Medical Sciences, Zhengzhou, Henan, China

**Keywords:** Xuanfu Hua, hepatocellular carcinoma, sorafenib, NOTCH1, FOXP3

## Abstract

**Background:**

It is crucial to explore ways to increase the sensitivity of hepatocellular carcinoma cells to sorafenib.

**Methods:**

The HepG2 and Huh7 cell lines with overexpressed HIF-2α were constructed. The cells were treated with Xuanfu Hua Tang (Xuanfu HT) containing serum and sorafenib separately and by using both of them, the cell viability and other cell biology functions were detected by CCK-8 and other assays. The mechanism of quercetin was investigated by thermal stability assay and dual luciferase reporter gene assay, and the effects of Xuanfu HT on the transcript and protein levels of Notch1 pathway genes were evaluated by qPCR and Western Blot. The effects of Xuanfu HT in tumor growth was investigates by mice subcutaneous tumor implantation model.

**Results:**

The Xuanfu HT increased sensitivity of HepG2 and Huh7 cell lines with overexpressed HIF-2αto sorafenib, and enhanced inhibition of cell proliferation, migration, invasion and angiogenesis by sorafenib. The component quercetin of Xuanfu HT containing serum could inhibit the binding between HIF-2α and the promoter of the transcription factor FOXP3 to inhibit the expression of FOXP3, so as to inhibit the activation of Notch1 pathway and angiogenesis. The expression of FOXP3 counteracted the decrease in Notch1 and VEGF expression, and angiogenic capacity induced by the combined treatment with Xuanfu HT and sorafenib. The tumor growth inhibitory effects of Xuanfu HT and sorafenib in mice were proved by constructing a subcutaneous tumor model.

**Conclusion:**

Xuanfu HT can increase sorafenib sensitivity of hepatocellular carcinoma cells by antagonizing the Notch1 pathway through quercetin-binding HIF-2α.

## 1 Introduction

Hepatocellular carcinoma (HCC) is one of the leading causes of cancer deaths worldwide (1). Every year, there are more than 905,000 new cases of HCC and 83,000 deaths, and HCC is considered the fourth leading cause of cancer-related deaths worldwide (2, 3). It’s worth noting that at least 50% of new cases of HCC are in China, of which approximately 60% are initially diagnosed at intermediate or advanced stages, with a 5-year survival rate of 5%-15% (3). At present, the treatments for hepatocellular carcinoma include liver transplantation and partial hepatectomy, radiotherapy, chemotherapy, interventional therapy, molecular targeted therapy, immunosuppressive agents, and traditional Chinese medicine (5, 6). As one of the first first-line drugs, the tyrosine kinase inhibitor sorafenib has a more precise action pathway and can be used in all stages of hepatocellular carcinoma. It has been widely used, but the apparent efficiency is low and the resistance rate is high. During the course of systemic therapy, the hepatocellular carcinoma cells can develop sorafenib resistance after continuous administration (4). Continuous use of sorafenib will up-regulate the expression of hypoxia-inducible factor 1α (HIF-1α) or hypoxia-inducible factor 2α (HIF-2α), thus promoting the transcription of genes such as mitochondrial phagocytosis, cell proliferation, glucose metabolism, angiogenesis, tumor invasion, and metastasis, and activating the Notch1 signaling pathway, which triggers cell resistance to sorafenib (5). According to relevant studies, HIF-2α can bind to the promoter of the transcription factor FOXP3 to increase FOXP3 expression, which in turn up-regulates the expression of Notch1 to activates the Notch1 signaling pathway, thus promoting angiogenesis (6). HIF has been described as an effective upstream regulator of different signaling pathways (7), which plays an important role in tumor neo-angiogenesis.

Xuanfu Hua Tang is a representative formula for the treatment of hepatocellular carcinoma by regulating qi and activating blood, which was created by Zhang Zhongjing, a famous doctor in Han Dynasty, in his *Synopsis of Golden Chamber* for the treatment of liver fixity. Many records written by later medical practitioners, and schools of thought showed that Xuanfu Hua Tang was effective in the treatment of liver diseases (8). The main components of the formula are monomers of inula flower (9) and Rubia cordifolia (10), which have significant efficacy in inhibiting the proliferation of hepatocellular carcinoma cells, cellular antioxidant, promoting apoptosis of hepatocellular carcinoma cells, and cellular autophagy. The inula flower contains multiple compounds, including quercetin, isoquercetin, caffeic-acid, chlorogenic-acid, inulin, and taraxasterol. Among them, quercetin (3, 3’, 4’, 5, 7-pentahydroxyflavone) is one of the main components of the flavonoid polyphenol family. In recent years, quercetin has demonstrated significant effects in the treatment of liver diseases (11), including improvement of metabolic functions, lowering of serum cholesterol, attenuation of inflammation and oxidative stress, inhibition of cell proliferation, and a variety of potential biological activities (12). Quercetin can inhibit the pathways promoting the development of hepatocellular carcinoma, including the signaling pathways (13) for inflammation, migration, apoptosis, fibrosis, and angiogenesis (14, 15). Previous studies demonstrated that the treatment combining quercetin/sorafenib significantly ameliorated liver injury (16, 17). However, there have been no specific reports on the mechanism by which Xuanfu Hua Tang increases sorafenib sensitivity of hepatocellular carcinoma cells.

To fill this gap, our work aims to investigate the mechanisms by which Xuanfu Hua Tang increases hepatocellular carcinoma cells ‘ sensitivity to sorafenib. According to our study, the component quercetin of Xuanfu Hua Tang bind with HIF-2α to inhibit the binding between HIF-2α and the promoter of the transcription factor FOXP3, so as to inhibit the expression of FOXP3. As a result, it can inhibit the activation of Notch1 pathway and suppresses cell proliferation, migration, invasion, and angiogenesis, thus increasing the sorafenib sensitivity of hepatocellular carcinoma cells.

## 2 Materials and methods

### 2.1 Cell culture

The human hepatocellular carcinoma cell lines HepG2 and Huh7 were purchased from the Chinese Academy of Sciences (Shanghai, China). The cells were cultured in Dulbecco’s Modified Eagle’s Medium (DMEM, Thermo, USA), added with 10% fetal bovine serum (FBS) (Beyotime, China) and 1% penicillin-streptomycin (Beyotime, China), and were incubated at in 5% CO_2_ at 37°C. Hypoxic conditions *in vitro* were simulated using CoCl2: following cell seeding and adherence, the cells were treated with CoCl_2_ (100 μM) and subsequently maintained under standard culture conditions for further experiments.

### 2.2 Preparation of Xuanfu Hua Tang aqueous extracts

Xuanfu Hua Tang (9g of Xuanfu Hua, 10g of Cong, 10g of Qianchao) was prepared according to the formula in *Synopsis of Golden Chamber*. After boiling Xuanfu Hua Tang with distilled water 3 times of its mass for 30 minutes, it was extracted twice; then, the combined extracts were concentrated by filtration to obtain the raw drug concentration of 4 g/mL, stored at 4°Cfor later use.

### 2.3 Cell transfection

The production and infection of retrovirus were performed as described previously (18). The pcDNA3.1-HIF-2α, pcDNA3.1-FOXP3 and packaging plasmid were transfected into the 293T cells using the Lipofectamine 3000 reagent (Invitrogen). After incubation, the virus particles were harvested for infection of hepatocellular carcinoma cell to construct the HIF-2α overexpression cell lines (OE HIF-2α) and FOXP3 overexpression cell lines (OE FOXP3). The untreated cells (Ctrl), and the cells transfected with pcDNA3.1 (OE Ctrl) were used as controls.

### 2.4 Immunofluorescence

Immunofluorescence (IF) staining was performed to detecting the expression of HIF-2α in HepG2 and Huh7 hepatocellular carcinoma cells under normal and hypoxic conditions. Cells were fixed with 4% paraformaldehyde, followed by antigen retrieval using Target Retrieval Solution (Dako, CA, USA). Subsequently, non-specific binding was blocked with 10% goat serum, and cells were incubated with a primary antibody (anti-HIF-2α, PA1-16510, anti-FOXP3, 11-5773-82, Invitrogen, Shanghai) at 4°C overnight. The next day, sections were washed with PBS and incubated with Alexa Fluor 488-conjugated goat anti-rabbit IgG secondary antibody (ab150077, Abcam, Shanghai) in the dark at room temperature for 1 hour. Nuclei were counterstained with DAPI (4’,6-diamidino-2-phenylindole). Finally, samples were mounted with anti-fade mounting medium, and fluorescence signals were visualized and captured under a confocal laser scanning microscope by examining five random fields. Five replicates were included for each cell type.

### 2.5 Cell activity and IC_50_ detection

The hepatocellular carcinoma cells in logarithmic phase were taken and inoculated into the 96-well plate with a cell density of about 5×10^4^ cells per mL, and 100 μL was inoculated in each well, and five replicates were set up for each cell. Then, the cells were placed in the incubator containing 5% CO_2_ for incubation at 37°C for 24h, 48h, 72h, and 96h, respectively. Next, 10 μL of CCK-8 solution (MBS2557034, MyBioSource, USA) was added to each well and incubated at 37°C for 2h, and the absorbance value at 450nm was measured using an enzyme calibrator (TECAN, infinite M200 pro), the measurements were repeated 3 times per cell. IC50: Referring to the method of Zhu et al. (19), after intervention of the cells using serum-containing Xuanfu Hua Tang and sorafenib for 48h, the CCK-8 solution was added, and the cell viabilities were detected by the CCK-8 method, and the sorafenib IC50 value of the cell line was calculated.

### 2.6 Western blot

Whole cell lysates were extracted by RIPA (VWRCN653-100ML, Amresco, USA) lysis buffer and centrifuged. The protein concentration was detected using the BCA protein assay kit (Thermo, 71285-M, USA). The samples were diluted in 5× SDS uploading buffer and separated by 10% or 12% SDS-PAGE; then, they were transferred onto polyvinylidene difluoride (PVDF) membranes (Millipore, USA), and sealed in 5% skimmed milk at room temperature for 1 h. Next, the membranes were incubated with the primary antibodies at 4 °C overnight, and then incubated with the secondary antibodies. The PVDF membranes were developed and exposed using ECL chemiluminescent chromogenic solution. Then, the Invitrogen iBright gel imaging system (Thermo, iBright CL1500, USA) was used to scan the samples, and grayscale analysis of the blot area was conducted by the ImageJ image analysis software. See **Table 1** for the antibody information. All experiments were performed in triplicate for reproducibility.

**Table 1:** Antibody information.

	Primary antibody	Secondary antibody
HIF-2α	Anti-HIF-2-alpha antibody (ab109616), Abcam, UK	Goat Anti-Rabbit IgG H&L (HRP) (ab205718), Abcam, UK, 1:1000
FOXP3	Recombinant Anti-FOXP3 antibody [EPR22102-37] (ab215206), Abcam, UK, 1:1000	
Notch1	Recombinant Anti-Notch1 antibody [EP1238Y] (ab52627), Abcam, UK, 1:1000	
VEGF	VEGF Antibody (MA1-16629), Thermo Fisher Scientific, USA	Goat Anti-Mouse IgG antibody (HRP) (ab205719), Abcam, UK, 1:1000
β-Actin	Anti-beta Actin antibody (ab8227), Abcam, UK, 1:1000	Goat Anti-Rabbit IgG H&L (HRP) (ab205718), Abcam, UK, 1:1000

### 2.7 Real-time fluorescence quantitative PCR

The relative expressions of related genes were detected by qPCR. The target gene sequences were obtained from the National Center for Biotechnology Information (NCBI) database, and the primers were designed using Snap Gene 4.2.4. RNA was extracted using the Eastep Super Total RNA Extraction Kit (Promega, LS1040, Madison, WI). cDNA was reverse transcribed from total RNA using GoScript™ Reverse Transcription Mix (Promega, A2790) according to the kit instructions. The PCR mixture was prepared using the Eastep^®^qPCR Master Mix kit (Promega, LS2062). Detection was performed using the real-time fluorescence quantitative PCR instrument (BIO-RAD, CFX96 Touch). The data were analyzed using the 2-^ΔΔCt^ method with β-actin as an internal reference. The primer sequences are shown in **Table 2**.

**Table 2:** Primer information.

Gene	Primer sequences
HIF-2α	F: 5´-TGAAAACGAGT CCGAAGCC-3´ R: 5´–GTGGCTGACTTGAGGTTGA-3´
FOXP3	F: 5´-TCACCTACGCCACGCTCAT-3´ R: 5´-ACTCAGGTTGTGCGGATGG-3´
Notch1	F: 5´-CCGTCATCTCCGACTTCATCT-3´ R: 5´-GTGTCTCCTCCCTGTrGTTCTG-3´
VEGF	F: 5´-GGCCAAAAACGAAAGCGCAAG-3´ R: 5´-GAGGCTCCAGGGCATTAGAC-3´
β-Actin	F: 5´- ATCTTCCGCCTTAATACT -3´ R: 5´- GCCTTCATACATCAAGTT-3´

### 2.8 Cellular thermal shift assay

The hepatocellular carcinoma cells were cultured for 8 h, and then exposed to DMSO for 24 h. Next, the cells were collected and washed with PBS containing protease inhibitor (1 mmol/L PMSF). The control cells were incubated using the same volume of PBS. After cell culture and counting, the cells were resuspended in PBS to a final density of 2×10^7^/mL. Then, the cells were dispensed into PCR tubes and heated in a thermal cycler (Bio-Rad, T100) for 3 min under a temperature gradient to denature the proteins. Next, the cells were resuspended in the NP40 buffer and subjected to 3 freeze-thaw cycles with liquid nitrogen. The supernatant was centrifuged and collected, and an equal amount of 2×SDS loading buffer was added to the supernatant, which was inactivated in boiling water for 10 min, and 20 μL of the supernatant was extracted. Then, Western blotting was performed using the SDS-PAGE gel.

### 2.9 Chromatin immunoprecipitation

ChIP was performed using the Pierce™ Magnetic ChIP Kit (26157, Thermo Scientific™). After being fixed with formaldehyde, the cells were collected. After ultrasonic cell-break, the antibodies (anti-HIF-2α) for target proteins were added to bind to the target protein-DNA complexes after ultrasonic break, and incubated overnight. Then, Protein A agarose was added to bind the antibody-target protein-DNA complexes, and after precipitation, the precipitated complexes were washed. After elution, the enriched target protein-DNA complexes were obtained. The enriched DNA fragments were purified after de-crosslinking, detection was performed by PCR.

### 2.10 Dual luciferase reporter gene assay

The cells were spread into the 6-well plate and cultured overnight, and the spreading density was based on the cell density that could reach 80% during transfection. The FOXP3 promoter fragment was cloned into the pGL3 luciferase reporter vector (Promega, Madison, USA), and the cells were co-transfected using HIF-2α plasmid. The fluorescence values were measured using the Dual-Luciferase^®^ Reporter (DLR™) Assay System (Promega, Madison, USA).

### 2.11 Angiogenesis assay

The cell culture medium was changed to serum-free DMEM medium. After culture for 48h, the cells were collected, centrifuged, and filtered, the tumor conditioned medium (TCM) was obtained, and the cells were added to the 48-well plates, three replicates were set up for each cell. After serum starvation treatment for 6 h, the human umbilical vein endothelial cells (HUVECs) were coated and inoculated with Matrigel (BD, Corning, USA), which were incubated at 37°C for 3 h. Then, the cells were treated with Chinese herbs. The angiogenesis of HUVECs at different time points was observed by microscopy.

### 2.12 Scratch assay

The hepatocellular carcinoma cells at logarithmic phase and the constructed cell lines were taken, and the cells were inoculated into the 6-well plate, with about 5×10^5^ cells in each well. On day 2, a straight line of uniform thickness was lightly scratched at the bottom of the 6-well plate with a 10 μL sterile tip using uniform strength at a uniform angle, and three replicates were set up for each group. The width of the scratch in the same field of view was observed and photographed under an inverted phase contrast microscope at 0 and 24 h, respectively. Measurements were repeated 3 times for each result. The healed area of the scratch was used to represent the cell migration ability.

### 2.13 Trans-well invasion assay

The matrix gel pre-cooled at 4 °C and diluted in 1:5 was added to the upper chamber of Trans-well, spread well and dried at 37 °C for 70 min, 5 replicate wells were set up in each group, and the cell concentration was adjusted to 5×10^5^ cells per ml. Then, 200 μL cell suspension was added to the upper chamber, and 500 μL of medium containing 10% FBS was added to the lower chamber as a chemotaxis factor. After adding the drug, the small chamber was cultured in 5% CO_2_ at 37 °C for 24 h. The un-transferred cells in the upper chamber were wiped off with a cotton swab, rinsed in PBS, fixed in methanol, and stained with 0.1% crystal violet. Next, 5 fields of view were selected for each well and photographed under an optical microscope, and the number of migrated cells in each group was counted.

### 2.14 Nude mice carrying tumor

The six-week-old female BALB/c-Nude mice were used in our study, which were purchased from SpePharm (Beijing) Biotechnology Co., Ltd. Ethical approval was obtained from the Henan Province Hospital of Traditional Chinese Medicine Ethics Committee before starting the study. The mice were weighed 23 g ± 2 g. They were fed at a temperature of 22 ± 1°C and relative humidity of 50-70%. The animals had free food and water intake. The more vigorous nude mice were selected and randomly grouped to prepare single-cell suspensions of hepatocellular carcinoma cells. The skin on the back of the nude mice was sterilized, and the single-cell suspension was injected into the subcutaneous right axillary dorsum of the nude mice. Each nude mouse was injected with the cell suspensions (about1×10^7^ cells). After injection, the mice in Xuanfu Hua Tang Group (n=5) were gavaged with aqueous extracts of Xuanfu Hua Tang (5 g/kg) once every 2 days for 4 weeks; the mice in Sorafenib Group (n=5) were injected intraperitoneally at a dose of 2 mg/kg sorafenib, once every 3 days; the mice gavaged with normal saline were used as the control group (n=5). About 7 days after the inoculation of cell suspension, obvious nodules could be detected under the skin at the inoculation site. The long and short diameters of the tumors were measured every 7 days, and the tumor volume was calculated: V=a*b^2^/2, where a is the long diameter of the tumor, and b is the short diameter of the tumor. The tumor growth curves were plotted based on the mean tumor volume of each group, and the nude mice were executed after 28 days to strip the tumors. The study was approved by the Henan Province Hospital of Traditional Chinese Medicine Laboratory Animal Ethics Committee (No. ACUC-2023NM041).

### 2.15 Statistical analysis method

The collected data were analyzed for significance and plotting using the GraphPad Prism 8 (San Diego, CA, USA) software. The measurement data were expressed as mean ± standard deviation (X ± S), the statistical differences between two groups were compared using t-test, and the comparisons among multiple groups were performed using one-way ANOVA. When p<0.05, it is considered as statistically significant.

## 3 Results

### 3.1 High expression of HIF-2α can increase the vitality of hepatocellular carcinoma cells and reduce their sensitivity to sorafenib

**Figure 1:**
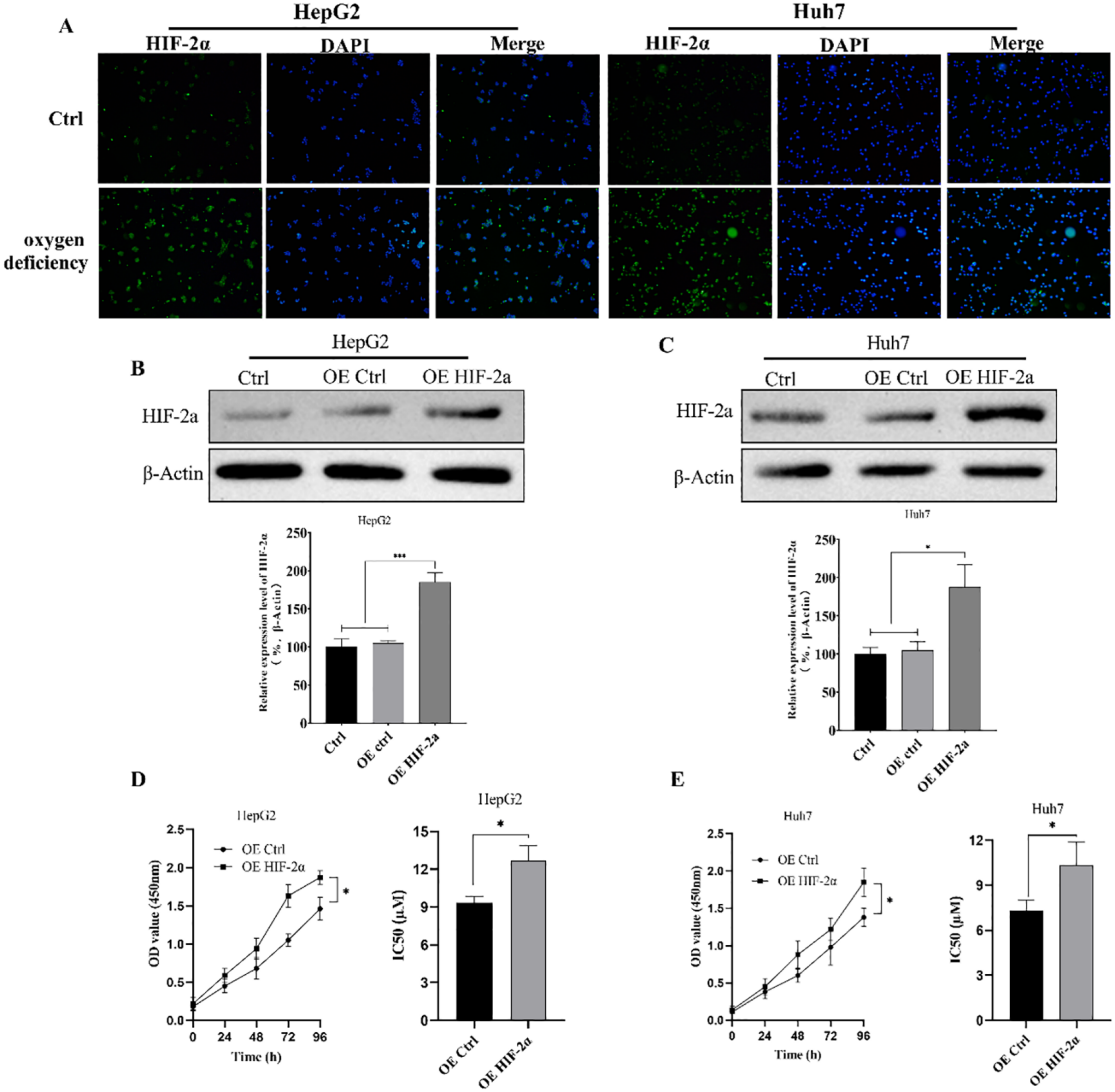
EEffect of overexpressed HIF-2α on cell viability and sorafenib sensitivity. **(A)** Differences in HIF-2α expression levels in hepatocellular carcinoma cells HepG2 and Huh7 in normal and hypoxic conditions detected by immunofluorescence; **(B, C)** Western Blot detection of HIF-2α expression levels in HepG2 and Huh7 cell lines with overexpressed HIF-2α; **(D, E)** Effect of overexpressed HIF-2α on sorafenib sensitivity in HepG2 and Huh7 cells as assessed by CCK-8 assay of cell viability changes and IC50 values in HepG2 cells with overexpressed HIF-2α. *p<0.05; ***p<0.001.

The hypoxia-inducible factor is a transcriptionally active protein with target genes related to hypoxia adaptation and tumor growth. To evaluate the HIF-2α expression difference of hypoxia-inducible factor in hepatocellular carcinoma cells in hypoxic environment, we detected the difference in HIF-2α expression levels of hepatocellular carcinoma cells HepG2 and Huh7 in normal and hypoxic conditions by immunofluorescence first, and the results were as shown in **Figure 1A**, in which the expression levels of HepG2 and Huh7 HIF-2αwas significantly increased in hypoxic conditions. Therefore, we further constructed the HepG2 and Huh7 cell lines with overexpressed HIF-2α (**Figures 1B, C**), and evaluated the cells’ sorafenib sensitivity by detecting the effect of overexpressed HIF-2α on cell viability and the IC50 value by CCK-8. According to the results, overexpression of HIF-2α significantly increased the viability of HepG2 cells, and overexpression of HIF-2α significantly reduced the HepG2 cells’ sensitivity to sorafenib. and the IC50 value of HepG2 cells was increased from 9.33 ± 0.51µM to 12.69 ± 1.17 µM (**Figure 1D**). The viability of Huh7 cells with overexpressed HIF-2α was also significantly increased, and the IC50 was increased from 7.32 ± 0.32µM to 10.3 ± 1.54µM (**Figure 1E**). The results indicate that the HIF-2α expression was up-regulated in HepG2 cells and Huh7 cells in a hypoxic environment, and overexpression of HIF-2α significantly increased the viability of hepatocellular carcinoma cells and reduced their sorafenib sensitivity.

### 3.2 Xuanfu Hua Tang increases hepatocellular carcinoma cells’ sensitivity to sorafenib

**Figure 2:**
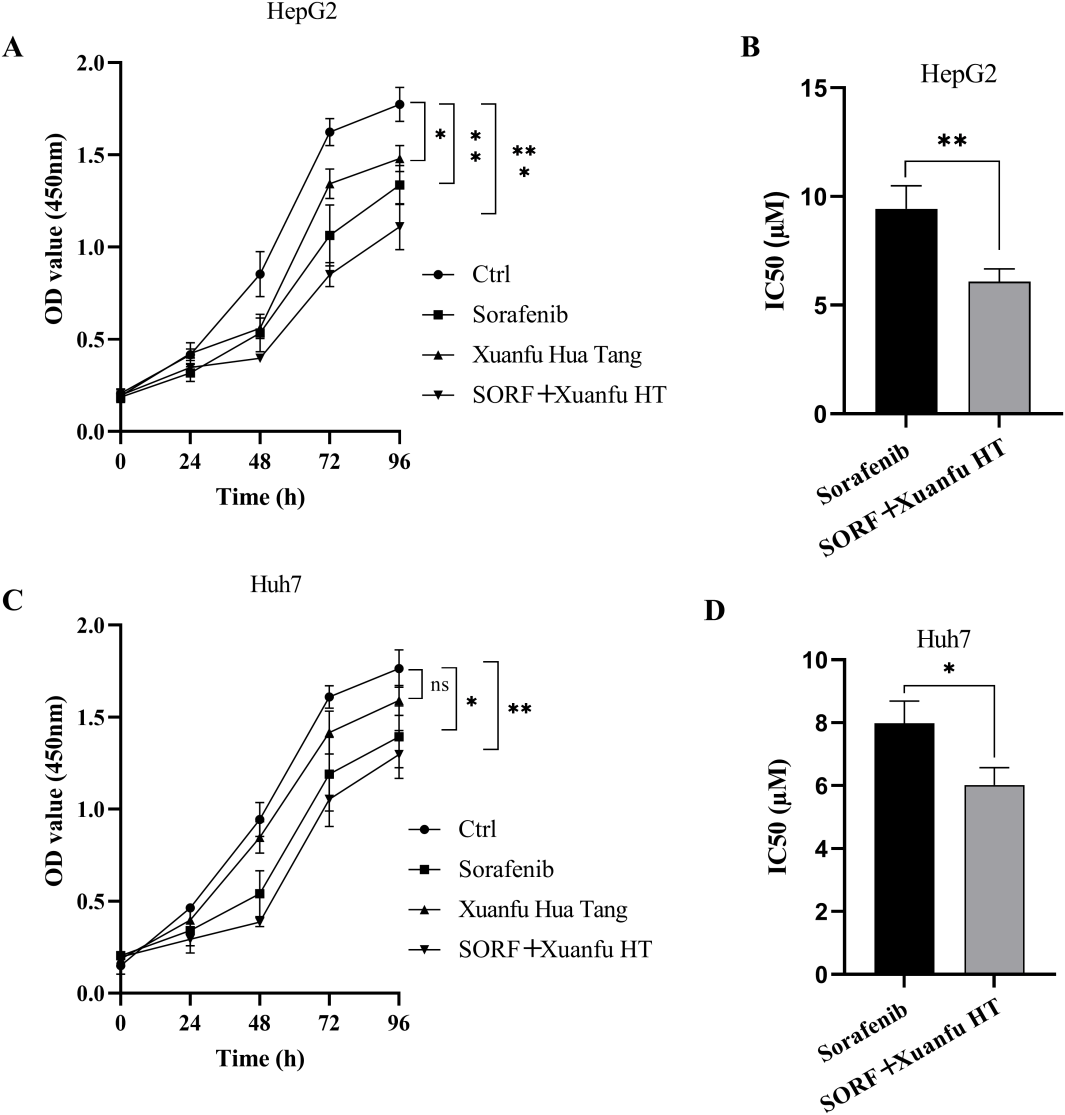
EEffects of serum containing Xuanfu Hua Tang on cell viability and sensitivity to sorafenib. **(A, C)** CCK8 detection of the viabilities of HepG2 and Huh7 cells with overexpressed HIF-2α cultured under different conditions: normal conditions, sorafenib, Xuanfu Hua Tang, and SORF + Xuanfu HT treatments; **(B, D)** Effect of Xuanfu Hua Tang on the sorafenib sensitivity of HepG2 and Huh7 cells with overexpressed HIF-2α according to the IC50 values. *p<0.05; **p<0.01; ***p<0.001; ns, no significant difference.

To investigate whether Xuanfu Hua Tang could inhibit the cell viability of HepG2 OE HIF-2αand Huh7 OE HIF-2α cells and increase their sensitivity to sorafenib, we treated the cells with serum-containing Xuanfu Hua Tang and sorafenib separately and in combination. The cell viability was detected by CCK-8 and analyzed for sorafenib sensitivity based on IC50 value. As shown in **Figure 2A** and **C**, the combination treatment of Xuanfu Hua Tang and sorafenib can better inhibit the cell growth than sorafenib alone. When only sorafenib was used, the IC50 of HepG2 cells detected by CCK-8 was 9.42 ± 1.07 μM; with the combined treatment of Xuanfu Hua Tang and sorafenib, the IC50 was 6.08 ± 0.59 μM (**Figure 2B**). Furthermore, when only treated with sorafenib, the IC50 of Huh7 cells detected by CCK-8 was 7.98 ± 0.71 μM, while the IC50 of cells treated with both Xuanfu Hua Tang and sorafenib was 6.02 ± 0.56 μM (**Figure 2D**). The results indicate that Xuanfu Hua Tang could significantly enhance the sensitivity of hepatocellular carcinoma cells to sorafenib.

### 3.3 Xuanfu Hua Tang can increase inhibition of migration and invasion of hepatocellular carcinoma cells by sorafenib

**Figure 3:**
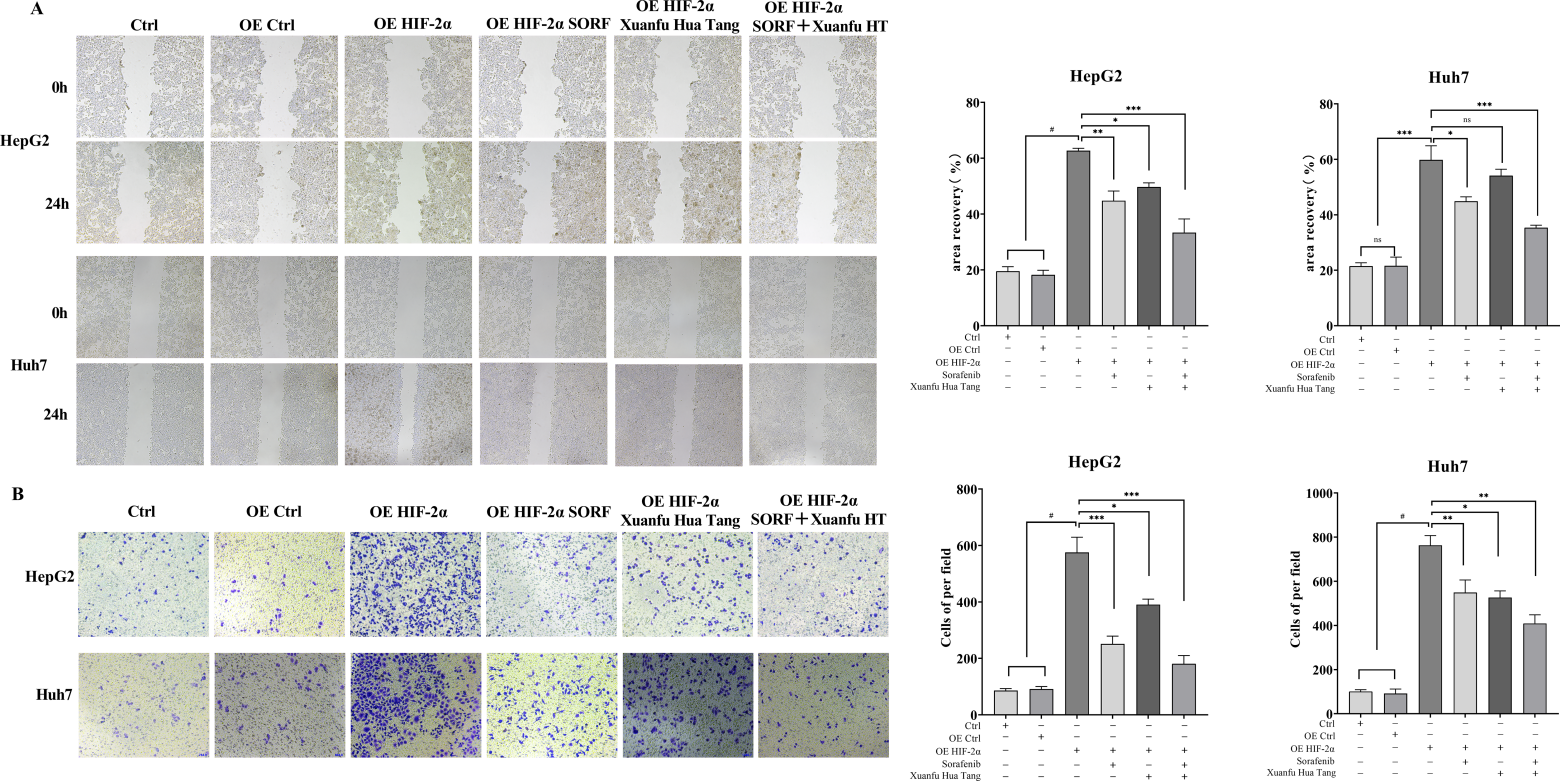
Effects of serum containing Xuanfu Hua Tang in combination with sorafenib on cell migration and invasion. **(A)** Scratch assay to detect the effects of sorafenib (SORF), Xuanfu Hua Tang (Xuanfu HT) and sorafenib + Xuanfu Hua Tang (SORF + Xuanfu HT) treatments on the migration of Huh7 and HepG2 cells with overexpressed HIF-2α; **(B)** Trans-well assay to detect the effects of sorafenib (SORF), Xuanfu Hua Tang (Xuanfu HT), and sorafenib + Xuanfu HT (SORF + Xuanfu HT) treatments on the invasion of Huh7 and HepG2 cells overexpressed HIF-2α. *p<0.05; **p<0.01; ***p<0.001; ^#^ *P*<0.0001.

To analyze the effects of Xuanfu Hua Tang on the physiology of hepatocellular carcinoma cells, we performed cell function analysis to evaluate the effects of Xuanfu Hua Tang combined with sorafenib on the migration and invasion of hepatocellular carcinoma cells Huh7 and HepG2 by scratch assay and Trans-well assay. The results of the scratch assay are shown in **Figure 3A**. Compared with the control, overexpression of HIF-2α in HepG2 and Huh7 cells significantly increased the cell mobility, and the combined treatment with Xuanfu Hua Tang with sorafenib significantly decreased cell mobility, which had a more significant effect than using sorafenib or Xuanfu Hua Tang alone. The invasion ability of cells was detected by Trans-well, and results indicate that the combination of Xuanfu Hua Tang with Sorafenib significantly reduced the increase in the invasion ability of HepG2 and Huh7 cells caused by HIF-2α overexpression (**Figure 3B**). The above results suggest that Xuanfu Hua Tang can increase the inhibition of cell migration and invasion by sorafenib.

### 3.4 Xuanfu Hua Tang can increase inhibition of angiogenesis by sorafenib

**Figure 4:**
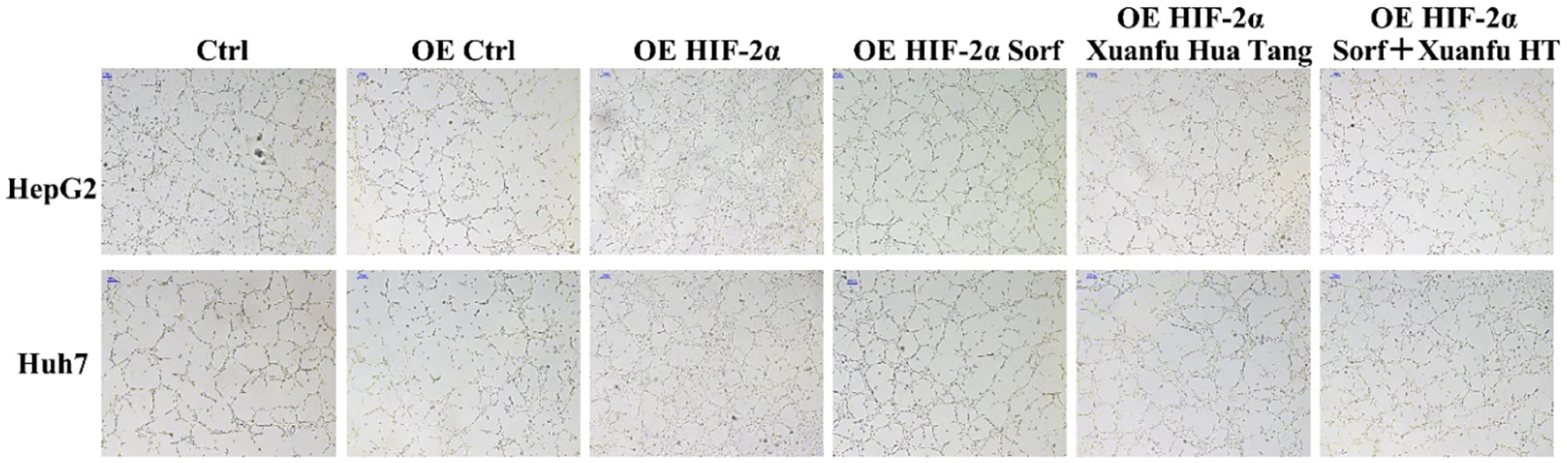
EEffects of Xuanfu Hua Tang treatment on angiogenesis inhibition by sorafenib. Effects of sorafenib and Xuanfu Hua Tang treatments separately and in combination on angiogenesis in HUVEC (100px).

HIF-2α plays an important role in tumor neo-angiogenesis. We tried to verify whether Xuanfu Hua Tang could increase sorafenib’s inhibition of angiogenesis caused by overexpression of HIF-2α in HepG2 and Huh7 cells. The results are shown in **Figure 4**. According to the results, after co-culture of the HepG2 and Huh7 cells with overexpressed HIF-2α, the HUVEC vascular growth was significantly increased, and the vascular network was tightly arranged and clearly visible. The treatment of cells with the serum-containing Xuanfu Hua Tang in combination with sorafenib significantly inhibited the blood vessel growth, and the blood vessels were sparse. The results indicated that Xuanfu Hua Tang increased sorafenib’s inhibition of angiogenesis induced by HIF-2α overexpression.

### 3.5 Serum-containing Xuanfu Hua Tang can inhibit Notch1 pathway via HIF-2α

**Figure 5:**
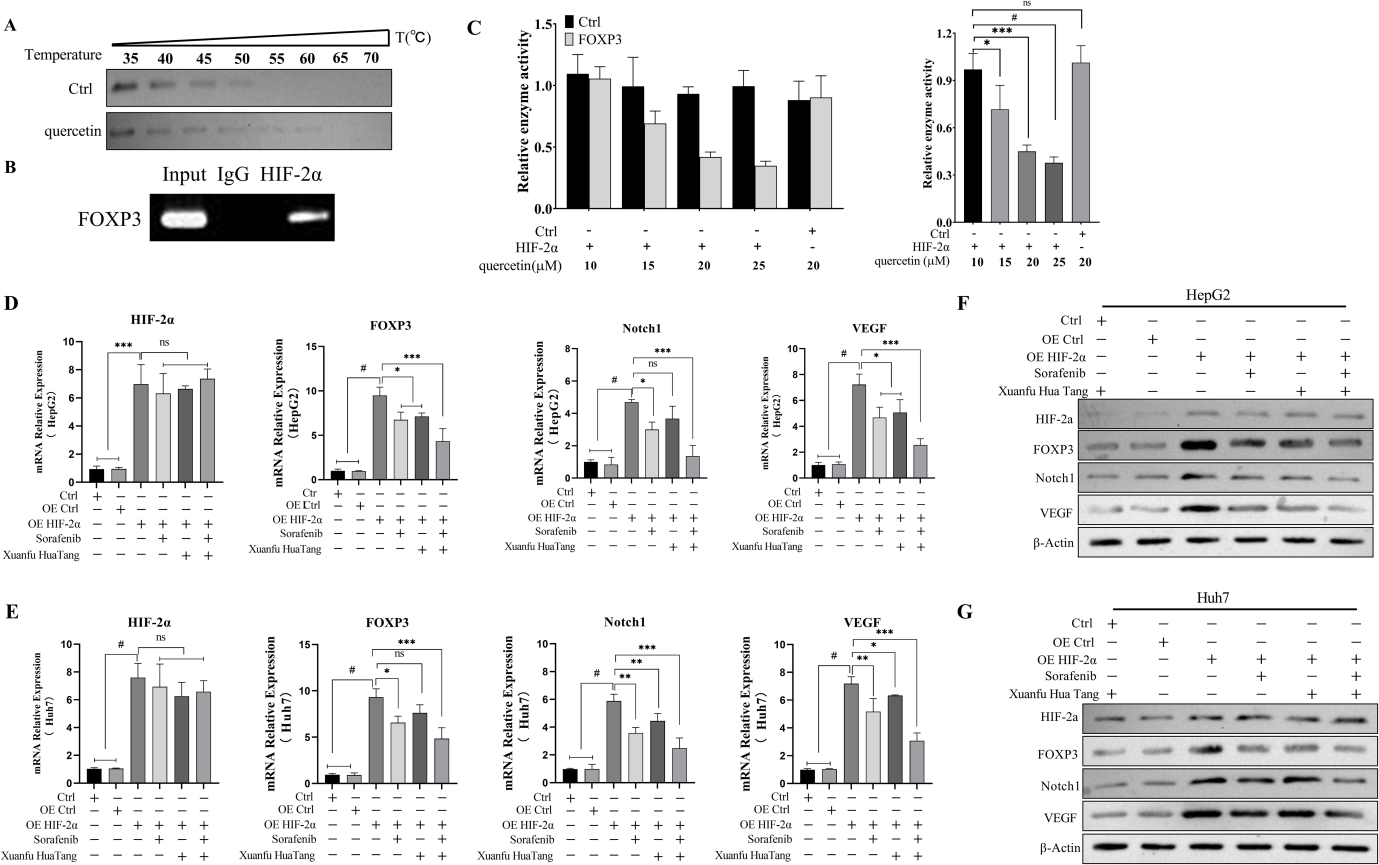
Quercetin inhibits the expressions of FOXP3, Notch1, and VEGF by binding to HIF-2α. **(A)** Binding of quercetin to HIF-2α determined by thermal stability experiments; **(B)** HIF-2α binding to the FOXP3 promoter detected by CHIP-PCR assay; **(C)** Dual luciferase reporter assay to analyze HIF-2α binding to the FOXP3 promoter; **(D, E)** qPCR detection of HIF-2α, FOXP3, Notch1 and VEGF mRNA transcript levels in HepG2 and Huh7 cells treated with Sorafenib, Xuanfu Hua Tang and Sorafenib + Xuanfu Hua Tang (SORF + Xuanfu HT); **(F, G)** Western blotting of the protein expression levels of HIF-2α, FOXP3, Notch1, and VEGF in HepG2 cells with overexpressed HIF-2α in the Sorafenib, Xuanfu Hua Tang, and Sorafenib + Xuanfu Hua Tang (SORF + Xuanfu HT) groups. *: p<0.05; **: p<0.01; ***: p<0.001; #: p<0.0001; ns, no significant difference.

Multiple signaling pathways are associated with the progression of hepatocellular carcinoma, among which HIF-2α is a key target of sorafenib. In order to investigate whether quercetin, a component of serum containing Xuanfu Hua Tang, also targets HIF-2α as its action point, firstly, we analyzed the action relationship quercetin and HIF-2α by thermal stability experiments, as shown in **Figure 5A**. In the experiments, with the increase of temperature, there was no obvious band in the control group, while there was a clear band in the quercetin treatment group, which indicates that HIF-2α bound to quercetin, so that it didn’t degrade with the increase of temperature. Furthermore, we preliminarily analyzed the HIF-2α-based action pathway of quercetin via CHIP-PCR and dual luciferase reporter assay. The CHIP-PCR assay showed (**Figure 5B**) that HIF-2α could bind to FOXP3 promoter. The luciferase activity assay showed (**Figure 5C**) that after transfection of plasmid with overexpressed HIF-2α, the transcriptional activity of FOXP3 was significantly decreased with the increase of quercetin concentration. To sum it up, this suggests that quercetin inhibited the expression of FOXP3 by targeting HIF-2α as its action point.

Then, we evaluated the effects of the serum-containing Xuanfu Hua Tang on the Notch1 pathway by q-PCR and Western blotting. In HepG2 and Huh7 with overexpressed HIF-2α, the transcript levels of the cellular transcriptional regulator FOXP3, Notch1, and vascular endothelial growth factor VEGF mRNA were significantly increased. The inhibitory effect of the Xuanfu Hua Tang combined with sorafenib on the transcriptional levels of FOXP3, Notch1, and VEGF mRNA transcript levels was more significant than that of sorafenib alone (**Figures 5D, E**). The Western blotting assay also verified that in the HepG2 and Huh7 cells with overexpressed HIF-2α, the protein expression levels of FOXP3, Notch1, and VEGF were significantly increased. Similarly, Xuanfu Hua Tang increased the inhibitory effect of sorafenib on the expression levels of FOXP3, Notch1, and VEGF (**Figures 5F, G**, the grayscale analysis statistics of WB are shown in **Supplementary Figure S1**). Additionally, we have conducted cell immunofluorescence experiments to evaluate the localization of FOXP3 under HIF-2α overexpression conditions. The experimental results demonstrate that upon HIF-2α overexpression, FOXP3 predominantly localizes in the cytoplasm rather than the nucleus (**Supplementary Figure S2**). In summary, these results demonstrate that quercetin could bind to HIF-2α to inhibit the binding of HIF-2α to the promoter of the transcription factor FOXP3, which in turn inhibits the expression of FOXP3, thus inhibiting the activation of Notch1 pathway and angiogenesis.

### 3.6 Xuanfu Hua Tang can intensify sorafenib’s reversal of angiogenesis induced by overexpression of FOXP3

**Figure 6:**
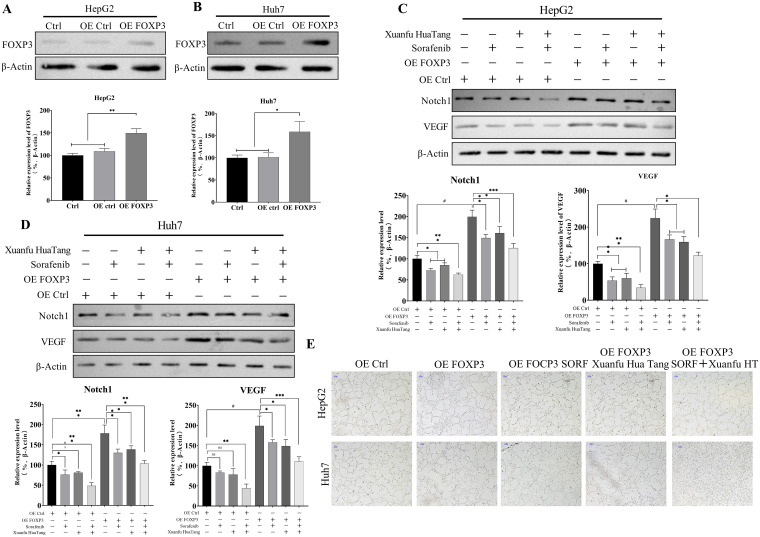
Reversal of angiogenesis caused by overexpression of FOXP3 using serum containing Xuanfu Hua Tang combined with sorafenib. **(A, B)** Western Blot assay for successful construction of HepG2 and Huh7 cell lines with overexpressed FOXP3; **(C, D)** Western Blot detection of the expression levels of Notch1 and VEGF in the HepG2 and Huh7 cells with overexpressed FOXP3 in Sorafenib, Xuanfu Hua Tang, Sorafenib + Xuanfu Hua Tang (SORF + Xuanfu HT) groups; **(E)** Co-culture with human umbilical vein endothelial cells (HUVEC) to detect angiogenesis. *p<0.05; **p<0.01; ***p<0.001; #p<0.0001.

To further prove that the component quercetin of Xuanfu Hua Tang can inhibit FOXP3 to inhibit the Notch1 pathway, thus suppressing neovascularization, we overexpressed FOXP3 in HepG2 and Huh7 cells, and treated the cells with serum-containing and Xuanfu Hua Tang and sorafenib. As shown in **Figures 6A** and **B**, HepG2 OE FOXP3 and Huh7 OE FOXP3 cells showed a significant increase in FOXP3 expression compared with the control group, and the cell lines were successfully constructed. The overexpression of FOXP3 in HepG2 and Huh7 cells resulted in a significant increase in the expression levels of Notch1 and VEGF, which offset the decrease caused by combined treatment of serum-containing Xuanfu Hua Tang and sorafenib (**Figures 6C, D**), but the Xuanfu Hua Tang increased the inhibition of Notch1 and VEGF expression levels by sorafenib in HepG2 and Huh7 cells with overexpression of FOXP3. Further, we co-cultured the cells with human umbilical vein endothelial cells (HUVEC) to detect angiogenesis. The overexpression of FOXP3 in HepG2 and Huh7 cells promoted angiogenesis, and Xuanfu Hua Tang enhanced the inhibition of angiogenesis by sorafenib (**Figure 6E**). In conclusion, the results further indicate that Xuanfu Hua Tang inhibited angiogenesis by suppressing the Notch1 expression.

### 3.7 Xuanfu Hua Tang can increase sorafenib’s inhibition of tumor growth in mice

**Figure 7:**
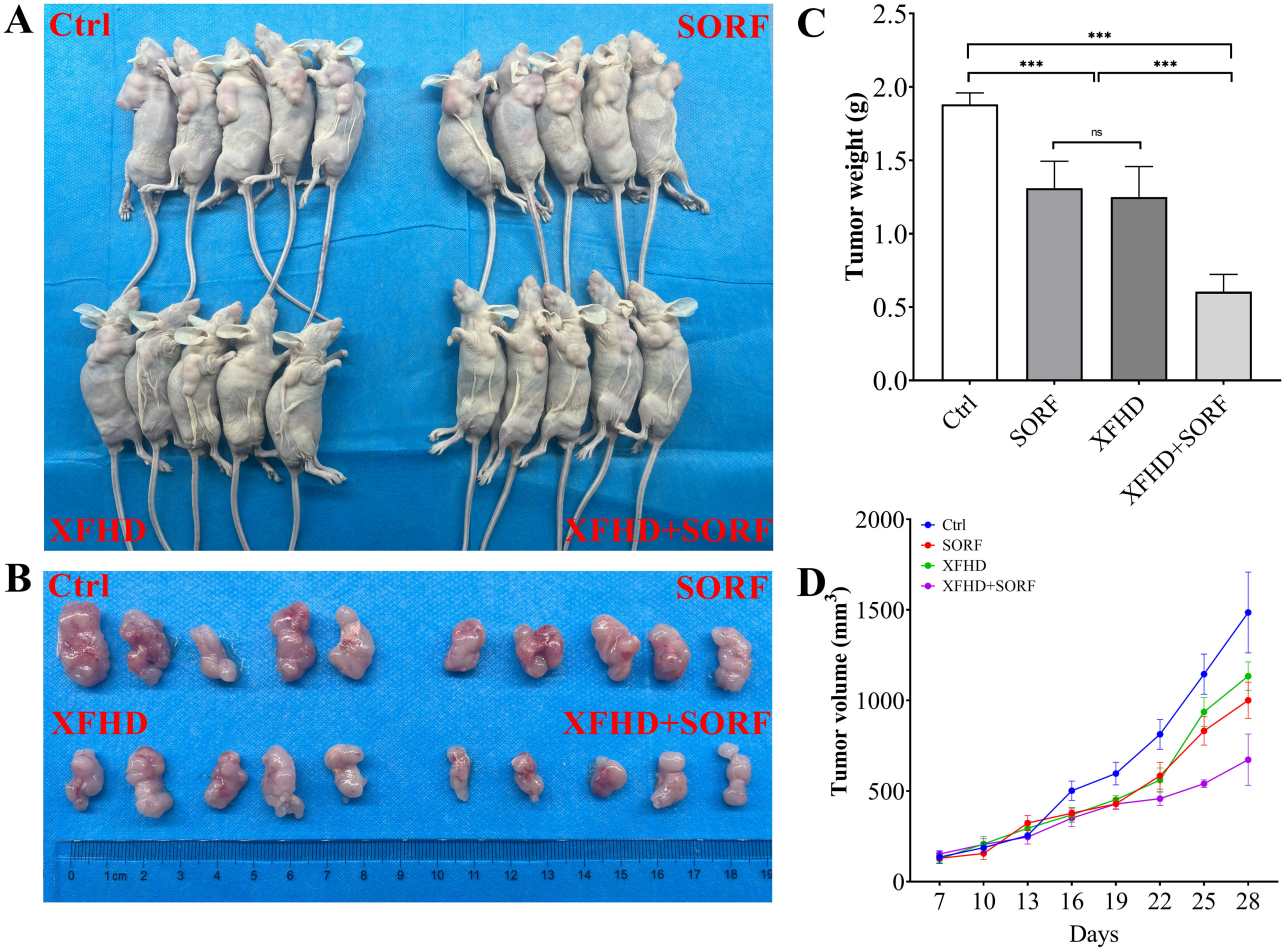
Xuanfu Hua Tang can increase sorafenib’s inhibition of tumor growth in mice. **(A)** nude mice photos (n=5); **(B)** the postoperative tumor photos; **(C)** tumor weight of nude mice; **(D)** tumor growth curves. ***p<0.001; ns, no significant difference.

To verify the effects of Xuanfu Hua Tang in mice, we constructed a mouse subcutaneous tumor implant model using HepG2 cells, and serum-containing Xuanfu Hua Tang and sorafenib were fed to the mice. The tumor sizes of mice were dynamically measured, and the tumor was removed and weighed at the 28^th^ day. The results, as shown in **Figure 7**, indicate that Xuanfu Hua Tang significantly increased the inhibition of tumor growth by sorafenib (**Figure 7A**), sorafenib treatment alone resulted in a tumor mass of 1.31 ± 0.184 g, and the combination of Xuanfu Hua Tang and sorafenib resulted in a tumor mass of 0.605 ± 0.118 g (**Figures 7B, C**). In terms of tumor size, the results were consistent with the trend of tumor weight (**Figure 7D**). Our results suggest that the Xuanfu Hua Tang increased sorafenib’s inhibition of tumor growth in mice.

## 4 Discussion

Sorafenib has been proved of being able to inhibit tumor cell proliferation and angiogenesis, which is a targeted therapeutic drug for advanced liver cancer (20, 21). However, due to multiple mechanisms promoting cellular resistance to sorafenib, it affects the treatment effects (22). Therefore, it is crucial to explore therapeutic strategies to improve sorafenib sensitivity. The traditional Chinese medicine (TCM) has been widely used in the treatment of hepatocellular carcinoma in clinical practice, and TCM alone or in combination with surgery has shown excellent efficacy (23). Zheng et al. demonstrated that TCM can improve the effects of target drugs and reduce resistance to targeted drugs (24). Continuous use of sorafenib can up-regulate the expression of hypoxia-inducible factor HIF-2α, promote the transcription of genes for mitochondrial phagocytosis, cell proliferation, glucose metabolism, angiogenesis, tumor invasion and metastasis, and activate the Notch signaling pathway, thus triggering resistance to sorafenib (5). This is consistent with our results, which showed up-regulated HIF-2α expressions of hepatocellular carcinoma cells HepG2 and Huh7 in hypoxic environment, and the overexpression of HIF-2α significantly increased cell viability and reduced sorafenib sensitivity.

Further, to investigate the efficacy of Xuanfu Hua Tang, we treated HepG2 and Huh7 cell lines with overexpressed HIF-2α with serum-containing Xuanfu Hua Tang and sorafenib separately and in combination, and the results showed that Xuanfu Hua Tang could enhance the sensitivity of HepG2 and Huh7 cells to sorafenib. Xuanfu Hua Tang is a representative formula for the treatment of hepatocellular carcinoma by the method of regulating qi and activating blood, and previous studies have shown that quercetin, a component of Xuanfu Hua Tang, exhibits different mechanisms of action at different stages of liver disease. It can inhibit the proliferation and spread of cancer cells through the signaling pathways related to hTERT, MEK1/ERK1/2, Notch, and Wnt/β-catenin in the advanced stages of hepatocellular carcinoma (25). Suzan Abdu et al. demonstrated (16) that when quercetin was used alone or in combination with sorafenib, it could significantly inhibit cancer cell growth, induce cell cycle arrest, apoptosis and necrosis, and down-regulate the expression of key inflammation, proliferation, and angiogenesis-related genes, which showed significant antioxidant and antitumor effects. Our findings further suggest that Xuanfu Hua Tang could increase the inhibition of angiogenesis and cell migration and invasion by sorafenib.

To investigate the action mechanism of Xuanfu Hua Tang, we examined the expression levels of related proteins. Interestingly, in HepG2 and Huh7 with overexpressed HIF-2α, the expression levels of the transcriptional regulator FOXP3, Notch1, and the vascular endothelial growth factor VEGF were significantly increased, and Xuanfu Hua Tang enhanced the suppression of FOXP3, Notch1, and VEGF by sorafenib. Therefore, we speculate that the action mechanism of Xuanfu Hua Tang is related to HIF-2α. We further demonstrated that quercetin, a component of Xuanfu Hua Tang, could bind to HIF-2α to inhibit the binding of HIF-2α to the promoter of the transcription factor FOXP3, so as inhibit the expression of FOXP3, which in turn could inhibit the activation of the Notch1 pathway, suppress angiogenesis, and increase the sensitivity to sorafenib. Sorafenib is an oral multikinase inhibitor targeting vascular endothelial growth factor receptors VEGFRl, VEGFR2, and VEGFR3, the platelet-derived growth factor receptor β (PDGFRβ), and Raf family kinases (26). Therefore, the inhibition of angiogenesis by Xuanfu Hua Tang could be related to the increased sensitivity to sorafenib. The Notch pathway is associated with tumor cell stemness, and blocking it signaling pathway can overcome drug resistance (27, 28). In addition, the Notch pathway is a classical signaling pathway with highly conserved structure, which is not only involved in the development of organisms and tissue regeneration, but also in the process of hepatocellular carcinoma development and progression, and it also plays an important role in promoting the invasion and migration of hepatocellular carcinoma. This is consistent with our findings that Xuanfu Hua Tang overcomes drug resistance by inhibiting the Notch1 pathway.

Then, we demonstrated the effects of combining serum-containing Xuanfu Hua Tang and sorafenib in mice by constructing a mouse subcutaneous tumor model with HepG2 cells, and the mice were fed with serum-containing Xuanfu Hua Tang and sorafenib. The tumor growth in mice can be inhibited by feeding Xuanfu Hua Tang and sorafenib alone, but the combined effect of the Xuanfu Hua Tang and sorafenib was more significant.

This study revealed that the combination of Xuanfu Hua Tang and Sorafenib can provide better drug properties. Compared with the effect of using only one of them, the combination of Xuanfu Hua Tang and Sorafenib was more effective in inhibiting cell proliferation, migration, invasion, and angiogenesis in HepG2 and Huh7 cells, and the cells were more sensitive to sorafenib. Therefore, Xuanfu Hua Tang can also be used as a reinforcing agent for sorafenib in the treatment of hepatocellular carcinoma. We also revealed that quercetin, a component of Xuanfu Hua Tang, could bind to HIF-2α to inhibit the binding of HIF-2α to the promoter of the transcription factor FOXP3, so as inhibit the expression of FOXP3, which in turn could inhibit the activation of the Notch1 pathway, suppress angiogenesis, and increase the sensitivity to sorafenib. The treatment combining Xuanfu Hua Tang and Sorafenib could be a potential therapeutic strategy for patients with hepatocellular carcinoma. However, the toxicity and clinical efficacy of this combination therapy remain to be evaluated, so further studies are required. Additionally, although this study found that HIF-2α overexpression is accompanied by changes in the total protein level of FOXP3, the exploration of its different isoforms and the mechanism of cytoplasmic retention remains insufficient. Moreover, since this study is primarily based on cell lines and mouse models, further investigations are warranted to explore the association between FOXP3 isoforms and subcellular localization in clinical hepatocellular carcinoma tissues. Such studies would provide a more comprehensive understanding of the regulatory network involved.

## Data Availability

The original contributions presented in the study are included in the article/**Supplementary Material**. Further inquiries can be directed to the corresponding author.

## Supplementary material

Supplementary Figure 1 WB grayscale analysis statistics of **Figure 5**. **(A)** the protein expression levels of HIF-2α, FOXP3, Notch1, and VEGF in HepG2 cells with overexpressed HIF-2α in the Sorafenib, Xuanfu Hua Tang, and Sorafenib + Xuanfu Hua Tang (SORF + Xuanfu HT) groups; **(B)** the protein expression levels of HIF-2α, FOXP3, Notch1, and VEGF in Huh7 cells with overexpressed HIF-2α in the Sorafenib, Xuanfu Hua Tang, and Sorafenib + Xuanfu Hua Tang (SORF + Xuanfu HT) groups. *p<0.05; **p<0.01; ***p<0.001; #p<0.0001.

Supplementary Figure 2 The localization of FOXP3 under HIF-2α overexpression and drug treatment. SORF, Sorafenib; XFHT, Xuanfu Hua Tang; SORF+ XFHT, Sorafenib + Xuanfu Hua Tang.
