# Supplementary figures and images for: Study on the mechanism by which Xuanfu Hua Tang increases sensitivity of hepatocellular carcinoma cells to sorafenib by antagonizing the Notch1 pathway through HIF-2α
Source: Front Oncol. 2025 May 15;15:1552480. doi: 10.3389/fonc.2025.1552480 (PMC12119620; doi:10.3389/fonc.2025.1552480)

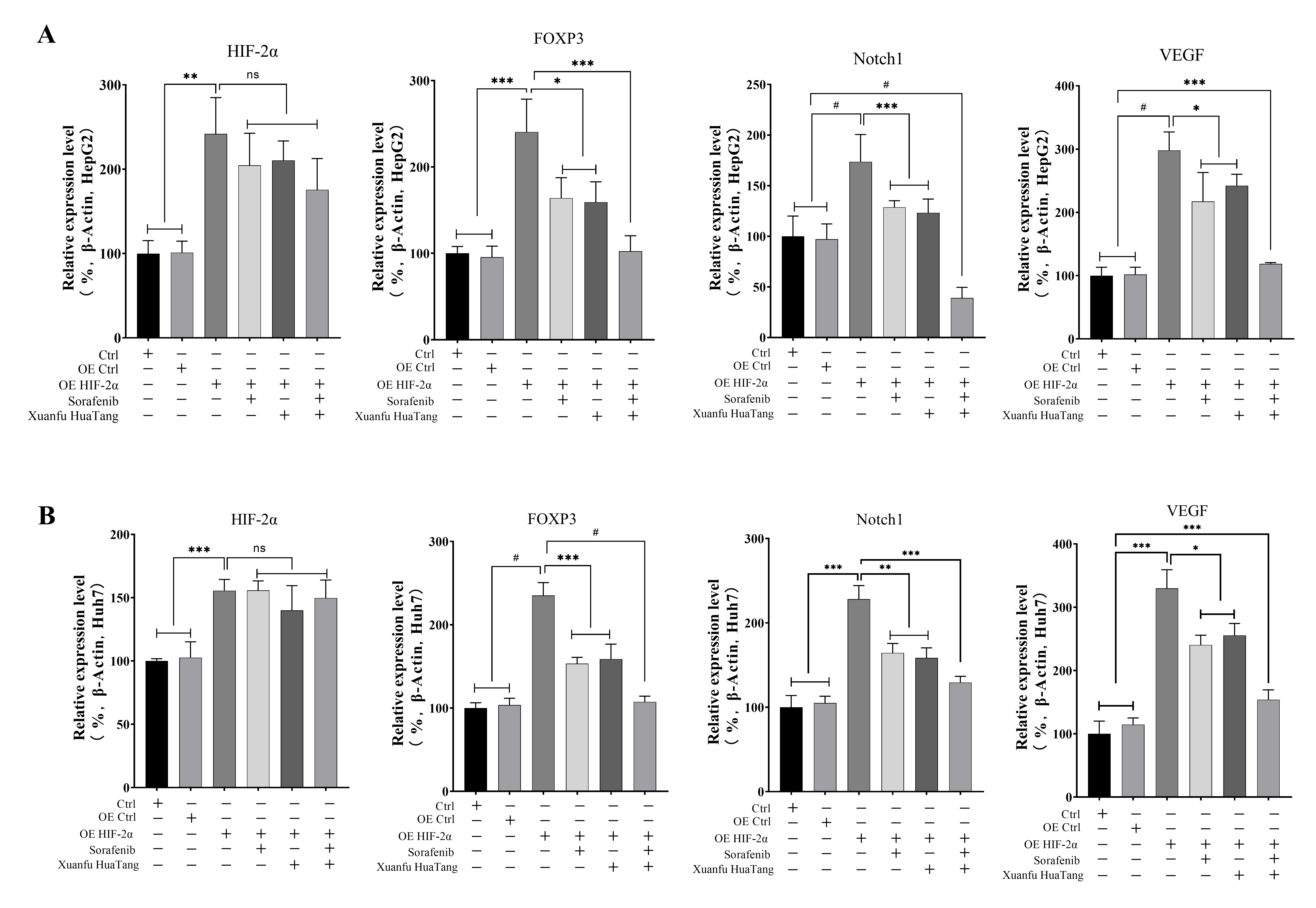

Supplement: Supplementary Figure 1 — WB grayscale analysis statistics of Figure 5 . (A) the protein expression levels of HIF-2α, FOXP3, Notch1, and VEGF in HepG2 cells with overexpressed HIF-2α in the Sorafenib, Xuanfu Hua Tang, and Sorafenib + Xuanfu Hua Tang (SORF + Xuanfu HT) groups; (B) the protein expression levels of HIF-2α, FOXP3, Notch1, and VEGF in Huh7 cells with overexpressed HIF-2α in the Sorafenib, Xuanfu Hua Tang, and Sorafenib + Xuanfu Hua Tang (SORF + Xuanfu HT) groups. *p<0.05; **p<0.01; ***p<0.001; #p<0.0001. [file Image1.tif]

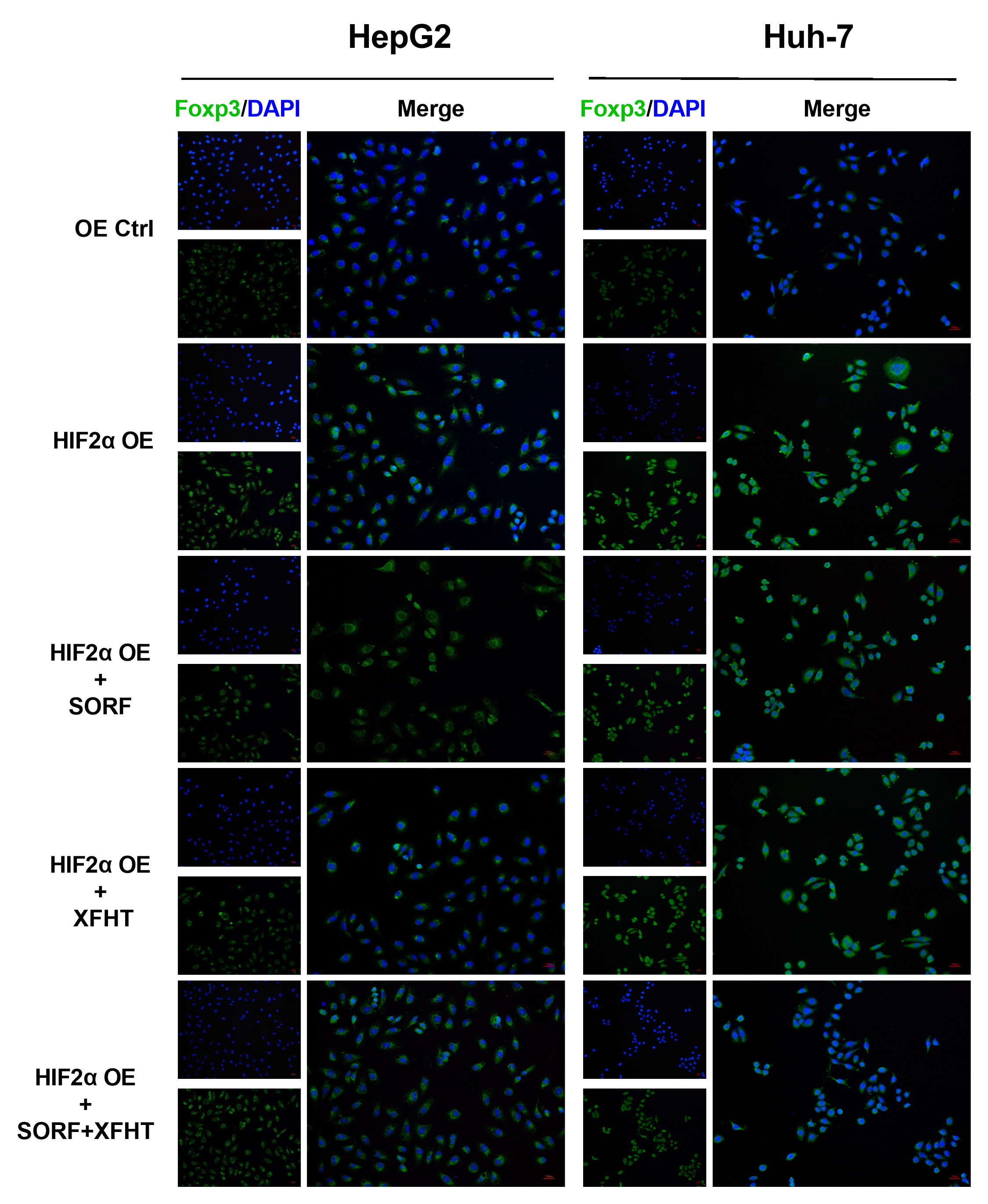

Supplement: Supplementary Figure 2 — The localization of FOXP3 under HIF-2α overexpression and drug treatment. SORF, Sorafenib; XFHT, Xuanfu Hua Tang; SORF+ XFHT, Sorafenib + Xuanfu Hua Tang. [file Image2.tif]
